# Identification of Key Genes and miRNAs in Osteosarcoma Patients with Chemoresistance by Bioinformatics Analysis

**DOI:** 10.1155/2018/4761064

**Published:** 2018-04-22

**Authors:** Binbin Xie, Yiran Li, Rongjie Zhao, Yuzi Xu, Yuhui Wu, Ji Wang, Dongdong Xia, Weidong Han, Dake Chen

**Affiliations:** ^1^Department of Medical Oncology, Sir Run Run Shaw Hospital, School of Medicine, Zhejiang University, Hangzhou, China; ^2^Department of Sports Medicine, School of Medicine, Zhejiang University, Zhejiang, China; ^3^Department of Surgical Oncology, Sir Run Run Shaw Hospital, College of Medicine, Zhejiang University, Hangzhou, Zhejiang 310016, China; ^4^Biomedical Research Center and Key Laboratory of Biotherapy of Zhejiang Province, Hangzhou, Zhejiang 310016, China; ^5^Orthopedic Department, Ningbo First Hospital, Ningbo 315000, China; ^6^Department of Urology, Wenzhou People's Hospital, Wenzhou, Zhejiang, China

## Abstract

Chemoresistance is a significant factor associated with poor outcomes of osteosarcoma patients. The present study aims to identify Chemoresistance-regulated gene signatures and microRNAs (miRNAs) in Gene Expression Omnibus (GEO) database. The results of Gene Ontology (GO) and Kyoto Encyclopedia of Genes and Genomes (KEGG) included positive regulation of transcription, DNA-templated, tryptophan metabolism, and the like. Then differentially expressed genes (DEGs) were uploaded to Search Tool for the Retrieval of Interacting Genes (STRING) to construct protein-protein interaction (PPI) networks, and 9 hub genes were screened, such as fucosyltransferase 3 (Lewis blood group) (FUT3) whose expression in chemoresistant samples was high, but with a better prognosis in osteosarcoma patients. Furthermore, the connection between DEGs and differentially expressed miRNAs (DEMs) was explored. GEO2R was utilized to screen out DEGs and DEMs. A total of 668 DEGs and 5 DEMs were extracted from GSE7437 and GSE30934 differentiating samples of poor and good chemotherapy reaction patients. The Database for Annotation, Visualization, and Integrated Discovery (DAVID) was used to perform GO and KEGG pathway enrichment analysis to identify potential pathways and functional annotations linked with osteosarcoma chemoresistance. The present study may provide a deeper understanding about regulatory genes of osteosarcoma chemoresistance and identify potential therapeutic targets for osteosarcoma.

## 1. Introduction

Osteosarcoma is one of the most common primary malignant bone tumors in children and adolescents. The worldwide morbidity rates of osteosarcoma are approximately with an average incidence of 3.1 per million for each stage and 4.4 per million for groups <25 years old. Additionally, there is a bimodal age distribution: individuals aged 25–60 years and elderly individuals, respectively. In America the Annual age-standardized incidence of osteosarcoma has reached stabilization from 1976 to 2005 [1–5].

Many factors are associated with tumor genesis, including high birth weight [6], pubertal hormones [7], and germline genetic variants [8, 9]. The common subtypes are osteoblastic, chondroblastic, and fibroblastic osteosarcomas, which may account for 70–80% of total cases [10]. The standard therapy consists of neoadjuvant chemotherapy (NACT), surgical removal of the primary tumor, and adjuvant chemotherapy. Before the 1970s, no more than 20 percent of patients were alive after 5 years when excision was major therapeutic measure for osteosarcoma [11, 12], while it increased to 60–70 percent for children and young adults with localized disease after the chemotherapy was used as adjuvant therapy for surgical resection [13, 14]. The current predicament of osteosarcoma treatment is the five-year survival rate does not exceed 25% for patients aged 2–68 with poor initial response tending to have adverse outcomes [15]. Therefore, to improve and modify chemotherapy regimens, an increasing number of pharmacogenomics studies on osteosarcoma have been going on for some time, such as drug reactions and toxicity.

Multidrug resistance protein 1 (MDR1) that is encoded by gene ATP-binding cassette, subfamily B (MDR/TAP), member 1 (ABCB1) has been shown to serve as a plausible factor in doxorubicin resistance, which was validated to be linked with poor outcomes in many osteosarcoma studies [16, 17], but whether there would be more valuable biomarkers remained to be explored. In recent years, microarray technology has substantially promoted the advance of understanding the mechanisms underlying diseases. Additionally, the rapid development of bioinformatics enables us to comprehensively screen out the hub genes associated with chemoresistance by the process of high-throughput microarrays. MicroRNAs (miRNAs), a group of highly conserved short noncoding small RNAs including generally 18–25 nucleotides in length, can suppress the translation of mRNA and cleave it by the modality of base-pairing to the target genes' 3′ untranslated region [18–20].

In the present study, we analyzed the data of GSE87437 and GSE30934 submitted by Serra Mand and Kobayashi E. et al., respectively, to get 668 differentially expressed genes (DEGs) and 5 differentially expressed miRNA (DEMs) between samples of poor and good chemotherapy reaction patients in GEO2R (http://www.ncbi.nlm.nih.gov/geo/geo2r/). To further understand the function of genes, Gene Ontology (GO), Kyoto Encyclopedia of Genes and Genomes pathway (KEGG), Protein-Protein Interaction (PPI) networks, and the connections among DEGs and DEMs were performed in sequence. We selected chemoresistance development related key genes and provided theoretical foundations for modifying and improving osteosarcoma treatment methods.

## 2. Materials and Methods

### 2.1. Microarray Data

The dataset of GSE87437, gene expression array, and GSE30934, miRNA expression array, included 10 and 8 samples from poor chemotherapy reaction patients and 11 and 16 samples from good ones, respectively. Moreover, the former dataset was based on GPL570 platform ([HG-U133_Plus_2] Affymetrix Human Genome U133 Plus 2.0 Array) and the latter one was based on GPL10312 platform (3D-Gene Human miRNA Oligo chip v12-1.00).

### 2.2. Identification of DEGs

GEO2R, an R-associated web application, was applied to filtrate DEGs between good chemotherapy reaction samples and poor chemotherapy reaction samples. In total, 21 samples in GSE87437 and 24 samples in GSE30934 were divided into two groups, respectively, and the concrete grouping schemes were already shown in microarray data. The *P* < 0.05 and |log⁡FC|*⩾* 1 were considered as cutoff criterion. All results of DEGs were downloaded in text format, hierarchical clustering analysis being conducted later in Morpheus (https://software.broadinstitute.org/morpheus/).

### 2.3. GO and Pathway Enrichment Analysis of DEGs

The online tool, Database for Annotation, Visualization and Integrated Discovery (DAVID, https://david.ncifcrf.gov/) provided comprehensive information for list of genes by GO and KEGG pathway analyses. In addition, GO enrichment analysis included three different aspects: biological process (BP), molecular function (MF), and cellular component (CC) [21]. KEGG enrichment analysis was associated with genomic information's functional interpretation and practical application [22]. The screened DEGs were uploaded to DAVID V6.8 to perform GO and KEGG pathway analysis with the criterion of *P* < 0.05, the results of which were downloaded in text format.

### 2.4. PPI Networks Construction and Module Analysis

To analyze the connection among proteins, DEGs were uploaded to Search Tool for the Retrieval of Interacting Genes (STRING, https://string-db.org/), a database covering 9,643,763 proteins from 2,031 organisms, and the result whose minimum interaction score was 0.4 was visualized in Cytoscape [23, 24]. Furthermore, the Molecular Complex Detection (MCODE) was used to screen out significant modules based on the constructed PPI networks with the criteria of degree cutoff = 2, node density cutoff = 0.1, node score cutoff = 0.2, *k*-core = 2, and max. depth = 100 and hub genes were exported. The functional enrichment analysis of genes in each module was performed in DAVID. Besides, the genes in each module were uploaded to DAVID and KEGG pathway enrichment analysis was conducted with the condition of *P* < 0.05.

### 2.5. Survival Analysis of Hub Genes

The series matrix of GSE21257 that contained osteosarcoma patients' prognostic information was downloaded from GEO database. The patients were split into two groups, high expression and low expression, according to the expression level of a specific hub gene. The data was processed by graphpad prism software and then exported the results.

### 2.6. Prediction of miRNA Targets

DEMs were acquired by the parallel method of DEGs mentioned above. miRWalk1.0 (http://zmf.umm.uni-heidelberg.de/apps/zmf/mirwalk/index.html), an integrated miRNA target prediction platform including 10 databases (DIANAmT, miRanda, miRDB, miRWalk, RNAhybrid, PICTAR4, PICTAR5, PITA, RNA22, and Targetscan), was utilized to explore the correlation between DEMs and DEGs. Besides, different colors were used to indicate the degrees of connections. For example, red color represented strong correlation.

## 3. Results

### 3.1. Identification of DEGs

A total of 668 DEGs were obtained from GSE87437 in the poor chemotherapy response samples and compared with those of good response with the criteria of *P* < 0.05 and |log⁡FC|*⩾* 1.0, comprising 422 upregulated genes and 246 downregulated genes. The key DEGs are displayed in Table 1.

### 3.2. Hierarchical Clustering Analysis of DEGs

**Figure 1:**
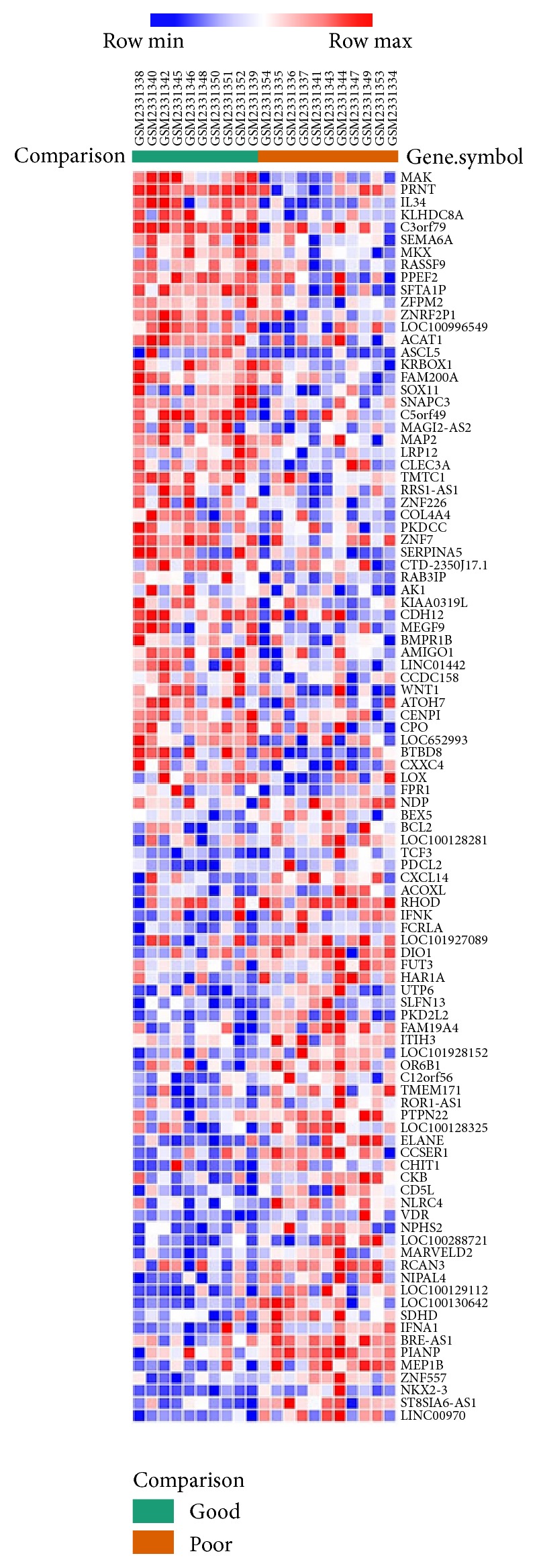
Heat map of the top 100 DEGs (50 upregulated genes and 50 downregulated genes). Red: up-regulation; Blue: down-regulation.

Hierarchical clustering analysis was conducted through Morpheus, a web-based online tool, with the series matrix data of the DEGs. The heat map is shown in Figure 1 (top 50 upregulated and 50 downregulated genes).

### 3.3. GO Term Enrichment Analysis

**Figure 2:**
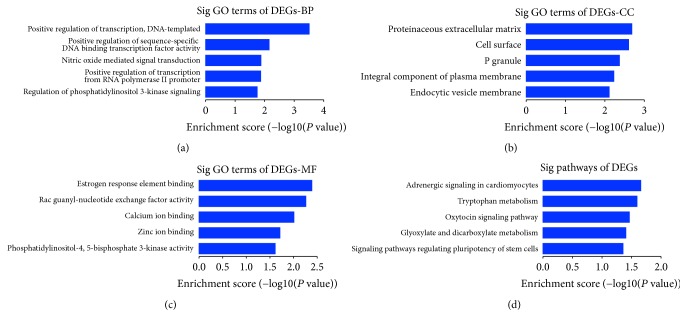
GO and KEGG pathway analysis of DEGs associated with osteosarcoma. (a) Top 5 significantly enriched biological processes in DEGs. (b) Top 5 significantly enriched cell component in DEGs. (c) Top 5 significantly enriched molecular function in DEGs. (d) Top 5 significantly enriched KEGG pathway in DEGs.

In order to understand the function of the identified DEGs deeply, GO and KEGG analyses were performed in DAVID, respectively. The result of GO analysis showed that DEGs were enriched in biological process (BP), including positive regulation of transcription and DNA-templated, positive regulation of sequence-specific DNA binding transcription factor activity, nitric oxide mediated signal transduction, positive regulation of transcription from RNA polymerase II promoter, and regulation of phosphatidylinositol 3-kinase signaling. As for molecular function (MF), the DEGs were enriched in estrogen response element binding, Rac guanyl-nucleotide exchange factor activity, calcium ion binding, zinc ion binding, and phosphatidylinositol-4,5-bisphosphate 3-kinase activity. Besides, Cellular Component (CC) analysis showed that the DEGs were enriched in proteinaceous extracellular matrix, cell surface, P granule, integral component of plasma membrane, and endocytic vesicle membrane, as shown in Figure 2.

### 3.4. KEGG Pathway Analysis

KEGG pathway analysis showed that DEGs were mainly involved in tryptophan metabolism, oxytocin signaling pathway, glyoxylate and dicarboxylate metabolism, cAMP signaling pathway, and dopaminergic synapse (Figure 2).

### 3.5. PPI Networks and Modules Selection

**Figure 3:**
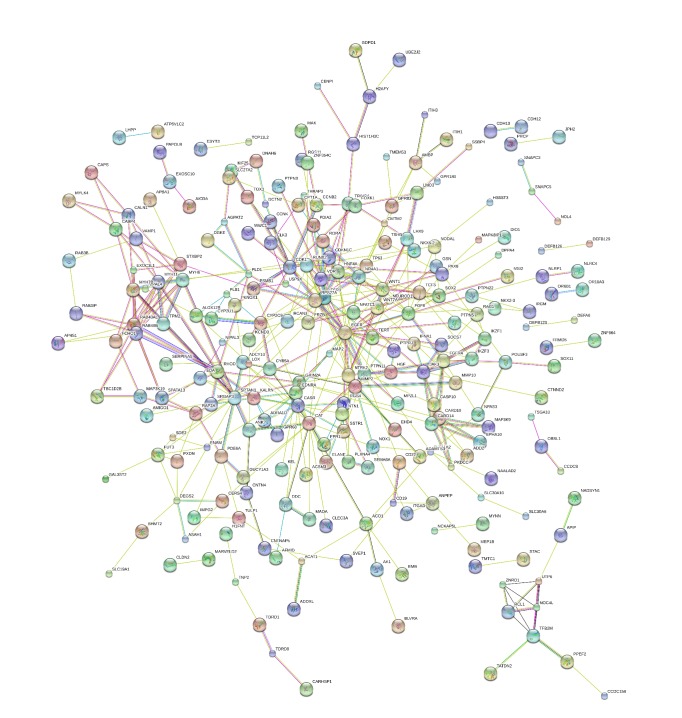
PPI network constructed by STRING database for the DEGs.

**Figure 4:**
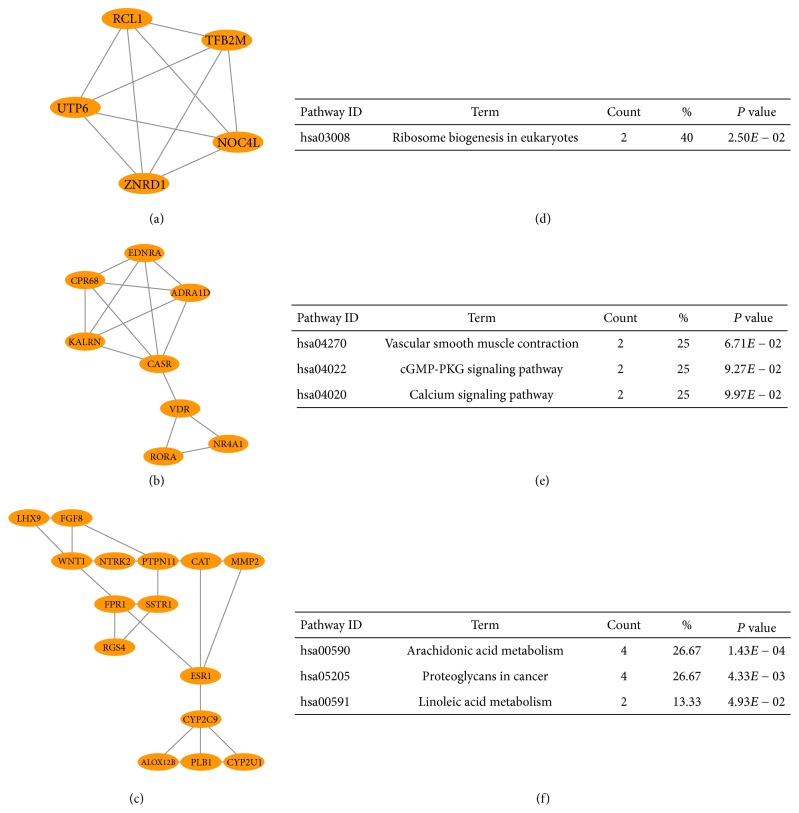
The top 3 modules from the PPI network. (a) module 1, (b) module 2, (c) module 3, (d) the enriched pathways of module 1, (e) the enriched pathways of module 2, and (f) the enriched pathways of module 3.

The PPI networks of DEGs were composed of 432 nodes and 428 edges (Figure 3). Then the networks were imported into Cytoscape software, analyzed by using plug-ins MCODE. Eventually, 3 significant modules were selected (Figure 4), and the KEGG pathway was mainly associated with ribosome biogenesis in eukaryotes, calcium signaling pathway, arachidonic acid metabolism, proteoglycans in cancer, and linoleic acid metabolism (Figure 4).

### 3.6. Hub Genes and Survival Analysis

**Figure 5:**
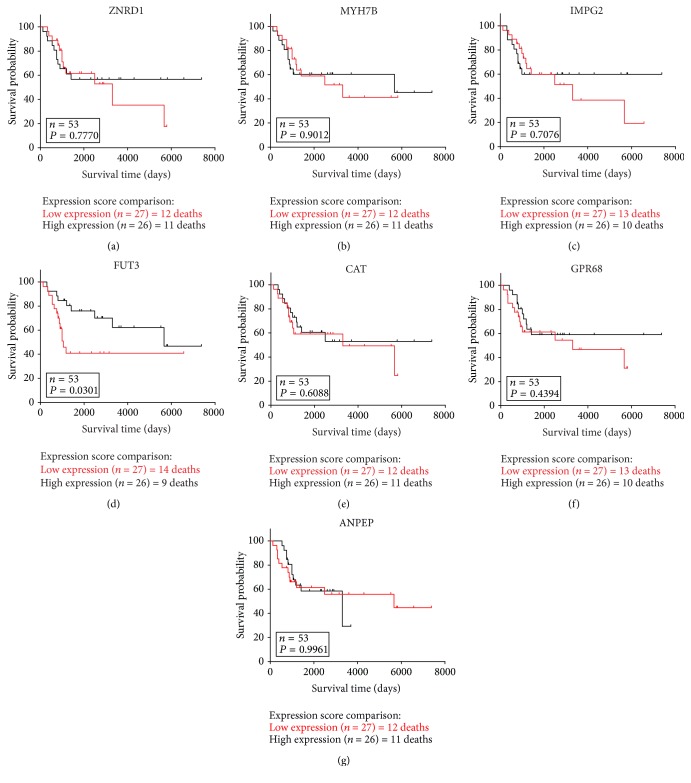
The survival prognostic value of hub gene in osteosarcoma from the GSE21257.

9 hub genes were screened out, including zinc ribbon domain containing 1 (ZNRD1), myosin heavy chain 7B (MYH7B), G protein-coupled receptor 68 (GPR68), catalase (CAT), fucosyltransferase 3 (Lewis blood group) (FUT3), interphotoreceptor matrix proteoglycan 2 (IMPG2), G protein-coupled receptor 180 (GPR180), alanyl aminopeptidase, membrane (ANPEP), and cyclin dependent kinase 1 (CDK1) (Table 1). Next, survival analysis of these genes in GSE21257 which contained patients' survival prognostic information showed that osteosarcoma patients with high mRNA expression of FUT3 meant a better overall survival (OS) despite its high expression in poor chemotherapy response samples (Figure 5). Additionally, the survival prognostic information of GPR180 and CDK1 was not included in GSE21257.

### 3.7. MiRNA–DEG Pairs

**Figure 6:**
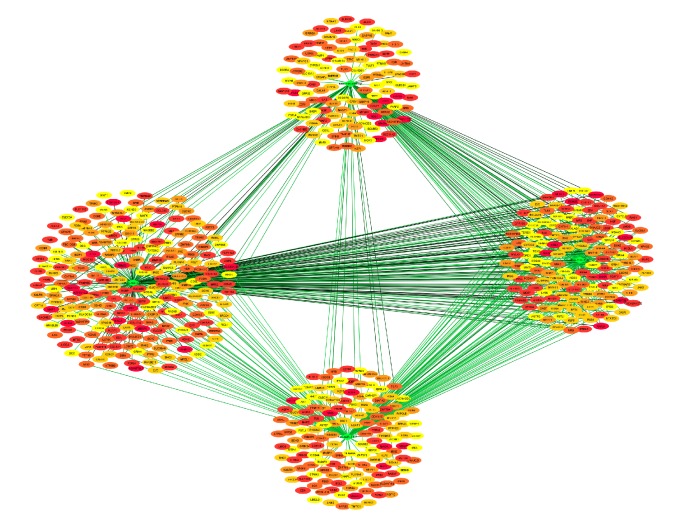
The network of miRNA–DEG pairs.

After the differentially expressed analysis for the data of GSE30934, a total of 5 DEMs were obtained between the poor chemotherapy response samples compared with that of good response with the criteria of *P* < 0.05 and |log⁡FC|*⩾* 1.0 (Table 2). Next, basing on miRWalk1.0 database (http://zmf.umm.uni-heidelberg.de/apps/zmf/mirwalk/index.html), the relationship between miRNAs and DEGs was acquired and different kinds of colors were on behalf of the number of miRNA–DEG pairs in different database which stand for the degrees of connection. For example, red color represented to a strong correlation (Figure 6). After comparing the targets with hub genes, we found that ZNRD1 was the potential target of hsa-miR-543, while CAT was the potential target of hsa-miR-518f. Both hsa-miR-543 and hsa-miR-518f matched the regulated gene in expression trends.

## 4. Discussion

In the present study, we observed whether there were more valuable genes like ABCB1 which could help improve and modify chemotherapy regimens in osteosarcoma. To find out the specific chemotherapy response-associated DEGs, we analyzed the osteosarcoma gene expression array of GSE87437 in GEO2R, where a total number of 668 DEGs were obtained between good and poor chemotherapy response samples. Besides, to further understand the potential biological functions, we conducted GO, KEGG, and STRING analyses. Subsequently, on the foundation of PPI networks, the selection of 9 hub genes and their survival prognosis were completed. In terms of the increasingly prominent role of miRNA in cancer, DEMs of osteosarcoma miRNA expression array of GSE30934 was screened out in the same way and criteria like DEGs, and DEGs-miRNA network was constructed to show relationship between them [25].

Our results showed that many genes and miRNAs may have functions in the development of chemoresistance in osteosarcoma and have the potential to become treatment targets. Here, we exclusively focused on 9 hub genes and two miRNAs. Firstly, 9 hub genes consisted of ZNRD1, MYH7B, GPR68, CAT, FUT3, IMPG2, GPR180, ANPEP, and CDK1. Our data showed that the expression of ZNRD1 was upregulated in chemoresistance osteosarcoma samples. Previous studies demonstrated that methotrexate-resistant, vincristine-resistant, multidrug resistant phenotypes of gastric cancer cells could be regulated by the inhibition of ZNRD1/Inosine monophosphate dehydrogenase 2 (IMPDH2), upregulated DARPP-32/downregulated ZNRD1, overexpressed miR-508-5p/ZNRD1/ABCB1 activities, respectively [26–28], but further researches of ZNRD1 in osteosarcoma chemoresistance remained to be conducted. Similar to ZNRD1, MYH7B was also found upregulated in chemoresistance osteosarcoma samples. At present, MYH7B was mainly involved in pathway of cardiomyocytes, such as mitochondrial apoptosis pathway [29] but studies about cancer were rare. GPR68, a kind of pH-sensing protein, was associated with tumor cell biology, such as tumor aggressiveness by triggering the intracellular signaling cascade to promote the development of microenvironment of extracellular acidification [30, 31]. As shown in Table 1, likewise, we found the expression of GPR68 was upregulated in chemoresistance samples. Daglioglu C validated that pH-responsive Fe3O4@SiO2(FITC)-BTN/QUR/DOX multifunctional nanoparticles could potentiate the chemotherapeutic efficacy of DOX against multidrug resistance as well as counteract the survival ability of chemoresistant lung carcinoma A549/DOX cell lines [32]. GPR68 has become an attractive target for drug development [33]. Several previous studies demonstrated that decreased CAT was highly associated with chemoresistance; for example, Xu et al. showed that intervention against miR-551b/CAT/reactive oxygen species (ROS)/Mucin-1 (MUC1) pathway might help overcome acquired chemoresistance [34]. Tumor microenvironment (TME) was characterized by hypoxia, acidosis, and dense extracellular matrix, providing tumors with resistance to various therapies, which could be effectively changed by the intravenous injection of human serum albumin (HAS)-chlorine e6 (Ce6)-CAT-paclitaxel (PTX) nanoparticles, enzyme-loaded therapeutic albumin nanoparticles. Meanwhile, H2O2 could relieve tumor hypoxia by generating oxygen within TME triggered by CAT of those nanoparticles, which made CAT a potential treatment target in various tumors [35]. Similarly, our data showed that CAT was downregulated in chemoresistance samples, which might exacerbate local microenvironment to strengthen tumor chemoresistance through the way of hypoxia, subsequent acidosis, and the like. High expression of FUT3 was proved to participate in the development of invasion, metastasis, and resistance to therapy by increased fucosylation activity in oral squamous cell carcinoma (OSCC), and the function could be blocked by inhibition of fucosylation [36]. In our study, FUT3 was also observed upregulated in chemoresistance samples, but it was found to be associated with a better survival prognosis (Figure 4). There were some reasons to explain that. In spite of its protumor action, death pathways were proved to be relied on fucosylation, and FUT3 was demonstrated to play an important role in natural killer-induced cytotoxicity after the recognition of sialyl Lewis X with the help of C-type lectin receptors [37, 38]. Therefore, the relationship between FUT3 and tumor was so complex that little was known about its function in osteosarcoma chemoresistance. IMPG-2, a gene mainly associated with retinal disease, was upregulated in chemoresistant osteosarcoma samples in our study, but more studies involving IMPG-2 and cancer needed to be conducted [39]. Furthermore, our data showed the decreased expression of GPR180 in poor chemotherapy response samples. Homoplastically, Honda et al. found that the methylation of GPR180 was probably to encode tumor suppressors and serves as a novel prognostic marker and therapeutic target in Hepatoblastoma [40]. Based on previous studies, whether the gene GPR180 could have function in osteosarcoma by producing tumor suppressors and its concrete role in chemoresistance remained to be explored. The gene ANPEP encoded aminopeptidase N (APN). A previous study [41] showed that ANPEP was downregulated in prostate cancer (PC). On the contrary, our study showed that ANPEP expression of good chemotherapy response samples was approximately two times that in chemoresistance osteosarcoma samples. However, the difference caused by the types of tumors or chemoresistance needed to be further studied. In urothelial carcinoma, APN could increase cytotoxicity of melphalan-flufenamide to play anticancer effect by amplifying the intracellular loading of melphalan [42]. The studies of chemoresistance associated with ANPEP have not been conducted so far, but Viktorsson et al. [42] offered researchers a new way for treatment, which made ANPEP a significant therapeutic target. Several researches in chemoresistance-associated fields have already demonstrated that CDK1 participated in the development of chemoresistance in pathways, such as GRP78/CDC2 [43]. Likewise, in our study, the expression of CDK1 was increased. Hayashi et al. found increased DNA repair activity in the G2-M transition promoted temozolomide (TMZ) resistance and CDC2 inhibitor flavopiridol (FP) treatment could resensitize TMZ-resistant clones in a p53-independent manner in glioma cells [44]. Besides, the combination of ERK inhibitor PD98059 and Taxol could improve the sensitivity of taxol-resistant tumor cells with the decreased CDC2 activity [45].

Compared with that of good response, 5 DEMs were acquired in GSE30934 in poor chemotherapy response samples (Table 2). Among them, hsa-miR-543 and hsa-miR-518f were found to have a relation to ZNRD1 and CAT, respectively. In our results, hsa-miR-543 was downregulated in chemoresistant samples. Previous study in this field was limited. In other aspects, decreased expression of it was involved in osteosarcoma angiogenesis which might be caused by connective tissue growth factor (CTGF) in phospholipase C (PLC)/protein kinase C (PKC*δ*) signaling pathway. Besides, hsa-miR-543 was also proved to be linked with tumor staging [46], cell proliferation, and the glycolytic pathway [47]. The studies between chemoresistance-promoted gene ZNRD1 and hsa-miR-543 have not been conducted yet, but biological functions mentioned above made hsa-miR-543 become an important therapeutic target. Moreover, the role of hsa-miR-518f in chemoresistance or the development of tumor was rarely known. Hsa-miR-518e and hsa-miR-518b, homologues of hsa-miR-518f, were demonstrated to be upregulated in hepatocellular carcinoma (HCC) [48]. Besides, a previous study showed that has-miR-518c-5p could regulate the growth and metastasis of oral cancer [49]. Consequently, further research to hsa-miR-518f was of great importance.

In summary, we identified 668 DEGs and 5 DEMs from GEO2R between good chemotherapy response samples and poor chemoresistance samples in osteosarcoma. And many of them, such as ZNRD1, GPR68, CAT, FUT3, ANPEP, CDK1, and hsa-miR-543, might be key genes related to osteosarcoma chemoresistance. These findings provided a series of promising treatment targets and enlightened us on the further investigations of the molecular mechanisms.

## Figures and Tables

**Table 1:** Key differentially expressed genes (DEGs) obtained from GSE87374.

Gene symbol	Log⁡FC	*P* value
ZNRD1	1.4	0.01504282
CDK1	1.31	0.03504221
MYH7B	1.19	0.03527194
GPR68	1.18	0.03724459
CAT	−1.15	0.03367398
FUT3	1.69	0.00007205
IMPG2	1.11	0.04785566
GPR180	−1.03	0.00511768
ANPEP	1.32	0.0354653

**Table 2:** Key differentially expressed genes (DEGs) obtained from GSE30934.

miRNA_ID	Log⁡FC	*P* value
hsa-miR-543	−3.429933	0.00192
hsa-miR-409-5p	−2.70517	0.00729
hsa-miR-518f	1.448711	0.02332
hsa-miR-154	−2.619116	0.03838
ebv-miR-BART1-3p	−1.358071	0.04733
